# New Holocene grey whale (*Eschrichtius robustus*) material from North Carolina: the most complete North Atlantic grey whale skeleton to date

**DOI:** 10.1098/rsos.220441

**Published:** 2022-07-20

**Authors:** Alyson Fleming, Briana Pobiner, Savannah Maynor, David Webster, Nicholas D. Pyenson

**Affiliations:** ^1^ Forest and Wildlife Ecology, University of Wisconsin Madison, Madison, WI 53706, USA; ^2^ Biology and Marine Biology, University of North Carolina Wilmington, Wilmington, NC 28403 USA; ^3^ National Museum of Natural History, Smithsonian Institution, Washington, DC 20560, USA; ^4^ Burke Museum of Natural History and Culture, University of Washington, Seattle, WA 98105, USA

**Keywords:** grey whale, Atlantic, Holocene, whaling, skeleton

## Abstract

Skeletal remains and historical accounts indicate that grey whales (*Eschrichtius robustus*) existed in the North Atlantic Ocean from the Pleistocene into the seventeenth century. Fossil and sub-fossil occurrences in this basin are rare, distributed from the east coast of the United States to Iceland and Europe. Here, we report an incomplete skeleton of a Holocene grey whale from Pender County, North Carolina, USA. This specimen represents a physically immature individual and is the most complete North Atlantic grey whale specimen reported to date. It comprises 42 cranial and postcranial elements, including the cranium, parts of the rostrum, both mandibles, both scapulae, humeri, radii and ulnae, most of the vertebral column anterior to the lumbar region and numerous ribs. Its provenance near the inlet of a large estuary is consistent with previous findings from the southeastern USA and parallels the species' habitat use in Baja California breeding and calving grounds in the North Pacific Ocean. Radiocarbon dating indicates an age of 827 ± 172 years before present. Cut marks on multiple skeletal elements indicate that the animal was butchered, suggesting some level of human exploitation of the species in the southeastern USA in the twelfth century, approximately 500 years prior to its extirpation in the North Atlantic.

## 1. Introduction

Grey whales (*Eschrichtius robustus* (Lilljeborg, 1867)) currently inhabit the North Pacific Ocean. However, the first published scientific descriptions of this species were based on sub-fossil remains from the North Atlantic collected from both the United Kingdom and Sweden in the early and mid-1860 s [1–4]. The descriptive work, based entirely on osteology, was published just a few years before Scammon [5] published observations of extant populations of the species in the North Pacific, to which Cope [6] designated the name *Rhachianectes glaucus*. More than two decades later, Lydekker [7] suggested that the grey whales in the two ocean basins were conspecific; near the mid-twentieth century, more rigorous osteological comparisons and nomenclatural revisions [8,9] led to the designation of *Eschrichtius* as the generic name for this species known in both the North Pacific and North Atlantic basins. Much of the nineteenth-century confusion surrounding the systematics of *E. robustus* (see [10]) has been the result of the fragmentary nature of the skeletal record from the North Atlantic basin, adjacent to centres of learning that were unaware of overlapping skeletal morphology elsewhere, either from sub-fossil remains or vouchers from the extant population in the North Pacific basin.

Since the first systematic work in the nineteenth century, more sub-fossil and fossil *E. robustus* material has been recovered, especially in the North Sea and along the eastern seaboard of the United States (electronic supplementary material, table S4). Fossil material referred to the genus *Eschrichtius* ranges in age from a 3.9–2.6 Ma fossil from the island of Hokkaido in Japan, to 44–40 ka fossils collected off the coast of Georgia in the USA (see [11] and [12]). Genetic analyses of North Atlantic specimens by Alter *et al*. [13] support a Pacific origin for the species, with most North Atlantic lineages nested within the genetic diversity of North Pacific grey whales, suggesting several dispersal events into the North Atlantic from the North Pacific during the Pleistocene.

Beyond systematic work, research to date on North Atlantic grey whale distribution, ecology and interaction with humans has all been limited to fragmentary remains, frequently single osteological elements. As a recent review by Charpentier *et al*. [14] explains, all whales have been ‘virtually invisible’ in the archaeological record. Highly pelagic in nature, recovered specimens are largely from strandings and active whaling events that bring whales to shore. Even then, the large size of whales results in beach and shore-line butchery, reducing their presence in archaeological records from middens and inland sites [15,16]. Bones found in such sites are frequently fragmented, minimizing potential for morphological identification [17]. However, the recent application of DNA analyses and collagen fingerprinting techniques has helped increase the number of large whale remains that can be identified to species [14,17,18].

Much of the interest surrounding North Atlantic grey whales concerns the cause and timing of their demise because both ethnographic and archaeological evidence points to an extirpation as recent as the seventeenth or eighteenth century (outside of extralimital individuals in the 2010s; see [19,20]). Through habitat modelling, Alter *et al*. [13] concluded that the range of suitable habitats for grey whales in the North Atlantic was extensive but that population sizes never reached those seen in the Pacific, and that diversity declined throughout the Holocene. The inferred antiquity from genetic evidence attributes the decline of North Atlantic grey whales to a historical time prior to intensive commercial whaling in the North Atlantic (approx. AD 1000). Prehistoric evidence for both opportunistic use and active whaling of North Atlantic grey whales exists but is limited. Historical accounts in the literature and archaeological evidence indicate that some level of whaling on grey whales did occur in Europe as well as on the northwest coast of the USA possibly as early as AD 800 and along the US east coast in the seventeenth century [10,17,21,22]. The zooarchaeological record has provided few insights into the nature and extent of human interaction with grey whales in the southeastern USA.

Here, we report on a new Holocene record of grey whale skeletal remains from Pender County, North Carolina, USA. This specimen comprises 42 cranial and postcranial elements including the cranium, parts of the rostrum, both mandibles, both scapulae, humeri, radii and ulnae, 19 vertebrae and 14 ribs, amounting to the most complete North Atlantic grey whale specimen yet reported. Radiocarbon dating indicates the age of specimen is 827 **±** 172 years before present. Many elements of the specimen have cut marks on them, indicating the animal was butchered. The specimen had been housed at the University of North Carolina Wilmington mammal collection since it was accessioned in 1987 when it was initially identified as humpback whale (*Megaptera novaeangliae* (Borowski, 1781)) osteology. At a later date, the species identity was reanalysed and confirmed as grey whale by Ann Pabst, Bill McLellan, Butch Rommel and author NDP (see ‘Diagnosis’ section below). Given the significance of the specimen, in 2021, the specimen was transferred to the collections at the Smithsonian's National Museum of Natural History in Washington, District of Columbia, USA. The completeness of the specimen offers opportunities for future morphological comparisons and investigations on the nature of human use of the species, half a millennium prior to its extirpation in this basin.

## 2. Material and methods

### 2.1. Institutional abbreviations

USNM and USNM PAL, Departments of Vertebrate Zoology and Paleobiology, respectively, National Museum of Natural History, Smithsonian Institution, Washington, District of Columbia, USA.

### 2.2. Description

Anatomical terminology follows Mead & Fordyce [23]. USNM PAL 706596 includes a largely intact cranium with incomplete parts of both maxillae and premaxillae, both mandibles, both major pectoral elements (i.e. scapulae, humeri, radii and ulnae), vertebrae C2–C7 and T1–T13, 11 right ribs (seven complete) and three left ribs. Research activities on the specimen were permitted through a Letter of Determination from NOAA covering these elements as MMPA pre-Act parts.

### 2.3. Butchery observations

Butchery marks were initially identified by NDP, documented by AF and SM and confirmed by BP via observations of photographs. Published criteria were used to identify butchery marks and distinguish them from other potential bone surface modifications [24,25].

## 3. Results

### 3.1. Systematic zoology

Cetacea Brisson, 1762

Pelagiceti Uhen, 2007

Neoceti Fordyce & de Muizon, 2001

Mysticeti Gray, 1864

Plicogulae Geisler *et al*., 2011

Balaenopteroidea Gray, 1868

Eschrichtiidae Ellerman & Morrison-Scott, 1951

*Eschrichtius* Gray, 1864 [2]

*Eschrichtius robustus* Lilljeborg, 1867

Type species. *Eschrichtius robustus* (Lilljeborg, 1867), by monotypy.

**Diagnosis.** USNM PAL 706596 belongs to *Eschrchtius robustus* based on the following differential diagnosis, which is based on Tsai & Boessenecker [26], in comparison with fossil and extant crown mysticete genera and species: *Eschrichtius* possesses mandibles with a low coronoid process unlike *Archaeschrichtius*, *Eschrichtioides*, *Megaptera* and species of *Balaenoptera*; mandibles lacking a prominent lateral curvature in the mandibular ramus anterior to the coronoid process (present in *Megaptera*, *Balaenoptera* spp. and Balaenidae); and mandibles lacking a patent, J-shaped mylohyoid sulcus on ventrolingual surface, known for all crown and stem Balaenidae. *Eschrichtius* possesses a narrow, arched rostrum, unlike *Pelocetus*, *Diorocetus* and *Parietobalaena*, *Megaptera*, and *Balaenoptera* spp.; frontals stepped ventrally relative to the dorsal level of the cranial vertex, as in *Megaptera*, and *Balaenoptera* spp., but with frontals widely exposed on vertex; large external bony nares relative to the width of the rostrum and with proportionally long and wide nasal bones, as in Balaenidae, and unlike *Eschrichtioides*; possessing a wider ascending process of premaxilla extending nearly to level of posterior termination of the nasal, unlike *Eschrichtioides*. Lastly, *Eschrichtius robustus* differs from *Eschrichtius akishimaensis* in having: an ascending process of maxilla that is narrow in dorsal view of the cranial vertex, occupying much less than the width of the premaxilla; possessing a posteromedially oriented border of the nasal, instead of a horizontally oriented one; and lacking deep and large squamosal concavity.

**Figure 1.**
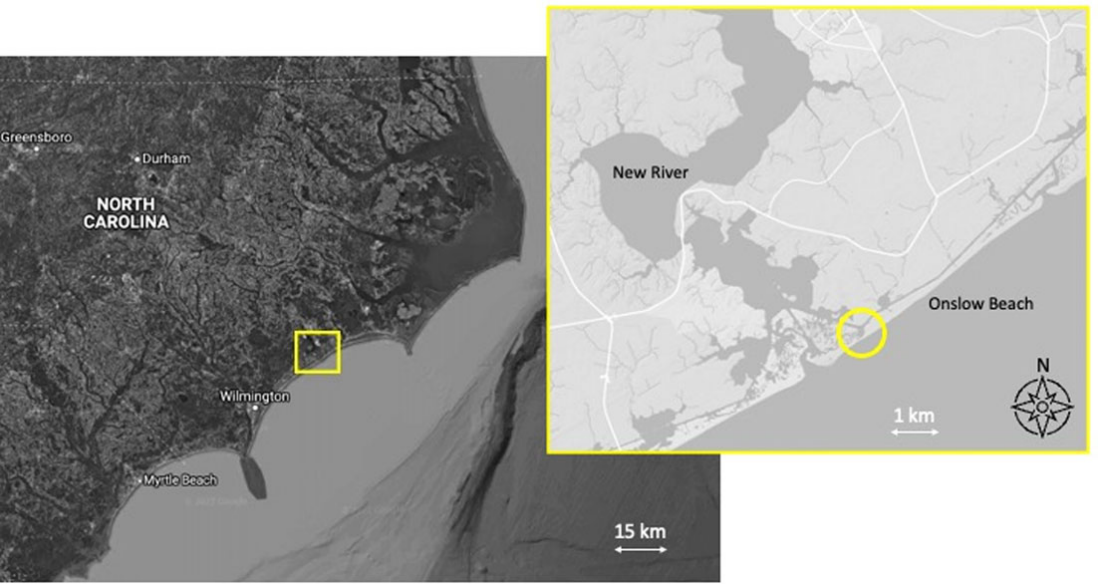
Map of specimen locality. Larger map shows the Carolina coast, with yellow box indicating the area of the inset map. Inset map shows New River inlet, with a yellow circle indicating the location where the specimen was found at the southwestern end of Onslow Beach.

**Locality.** The skeleton was recovered over the course of several years, probably in the 1970s, by the late Rita and Tom McCabe (Jacksonville, North Carolina) from the intertidal sands at the southwestern edge of Onslow Beach near New River Inlet, Onslow County, North Carolina, USA (figure 1). It was then donated to University of North Carolina Wilmington in 1987 and accessioned into the Smithsonian's collection as specimen USNM PAL 706596 in 2021. Field observations (AF, 2021) suggest that the likely collection location of the McCabes was approximately within 1 km of 34.543° N, −77.313° W. Root markings on the bone, consistent with *Spartina* and other wetland species, suggest the specimen was deposited in a marsh environment, partially submerged for some time, with the left side exposed (see Osteological description below).

**Age.** We used a sample from a rib bone for radiocarbon dating at the Keck Carbon Cycle AMS Facility in the Department of Earth System Science at the University of California, Irvine. The uncorrected radiocarbon age is 1360 ± 15 BP (14C years before AD 1950). Calibration was carried out using Calib 8.2 software with the Marine20 database [27] and a regional Δ*R* of −85 ± 54 14C years based on likely northeastern Atlantic grey whale feeding grounds and yielded a 2 sigma (95% confidence) calibrated age range of 655–1004 calendar years BP, with a median probability of 827 calendar years BP. See electronic supplementary material, table S1 for more details.

### 3.2. Osteological description

#### 3.2.1. Overview

Anatomical terminology follows Mead & Fordyce [23]. In most cases, the description of individual elements derives from the most informative side of the skull, in terms of preservation; we note any morphological asymmetry if present.

#### 3.2.2. Skull (cranium and mandibles)

**Figure 2.**
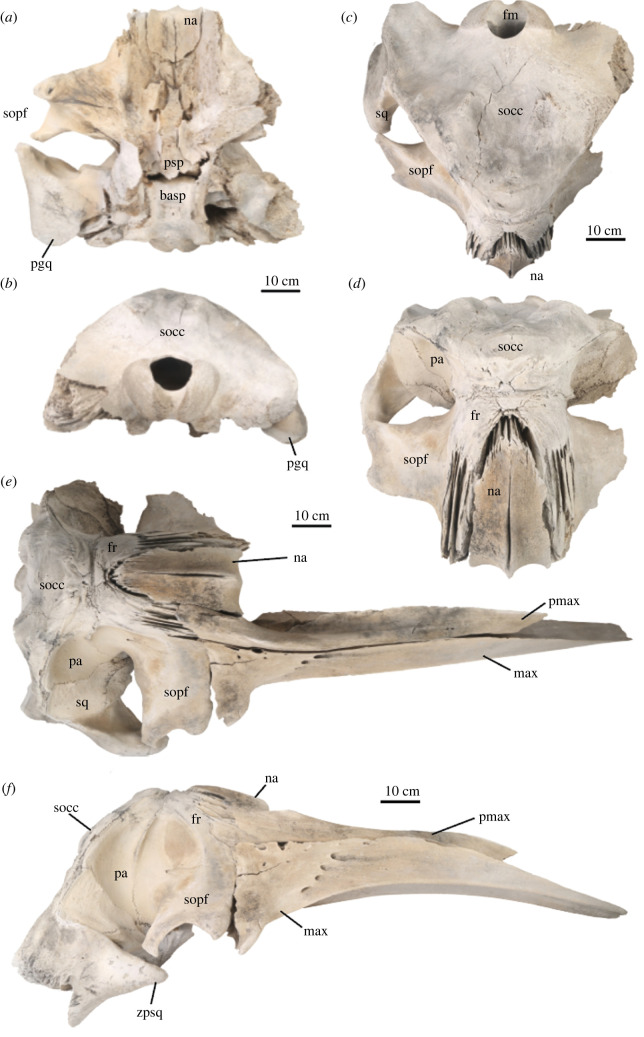
Cranium of USNM PAL 706596, *Eschrichtius robustus*. Cranium in (*a*) ventral view, (*b*) posterior view, (*c*) dorsal view and (*d*) oblique anterodorsal views. The cranium with the rostrum, including right maxilla and premaxilla in articulation, in (*e*) dorsal and (*f*) right lateral views. Anatomical abbreviations: basp, basisphenoid; fm, foramen magnum; fr, frontal; max, maxilla; na, nasal; pa, parietal; pgp, postglenoid process; pmax, premaxilla; psp, presphenoid; socc, supraoccipital shield; sopf, supraorbital process of the frontal. Original image credits: Courtney Johnson.

**Figure 3.**
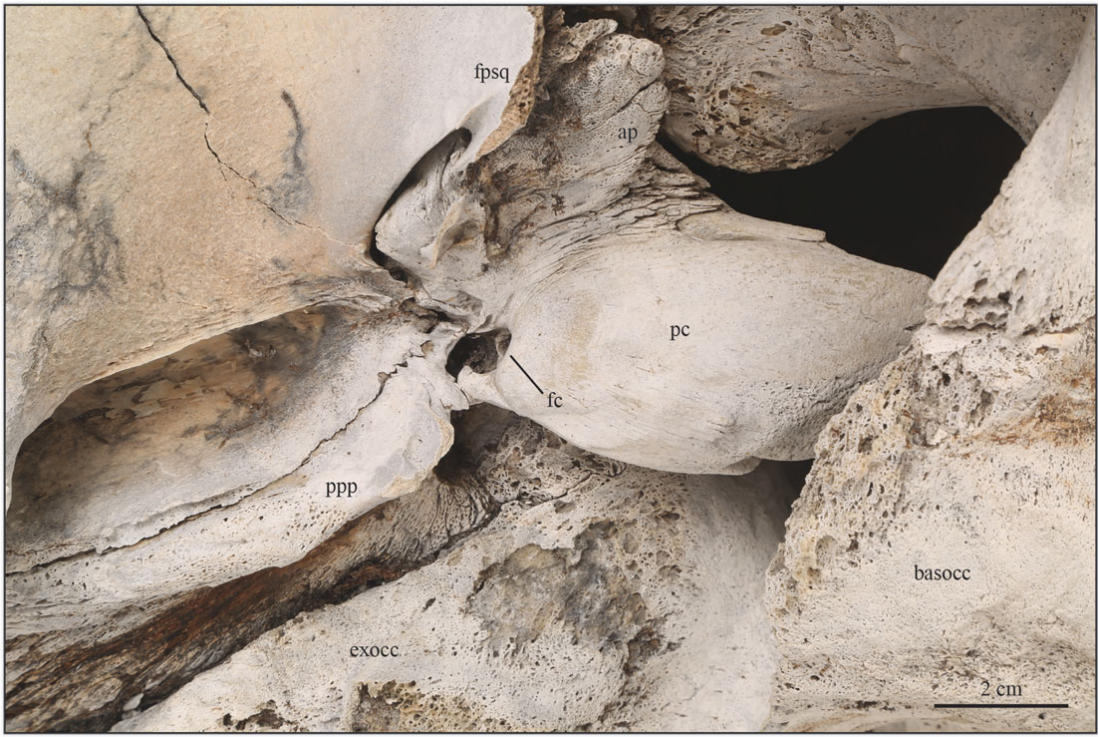
Right periotic of USNM PAL 706596, *Eschrichtius robustus in situ*, in ventral view. Abbreviations: ap, anterior process of the periotic; basocc; basioccipital crest; exocc; exoccipital; fc, fenestrae cochleae; fpsq, falciform process of the squamosal; pc, pars cochlearis; ppp, posterior process of the periotic. Original image credits: Courtney Johnson.

**Figure 4.**
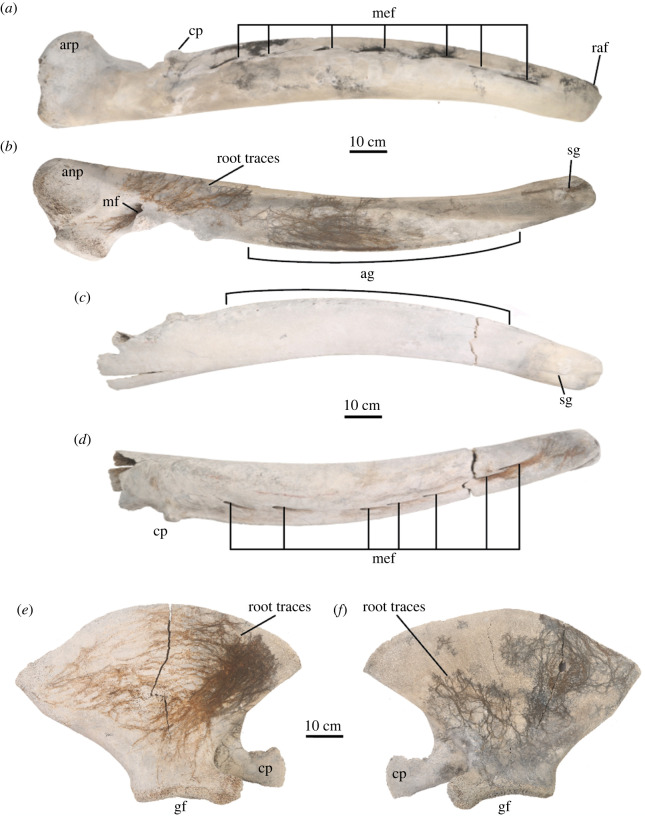
Mandibles and scapulae of USNM PAL 706596, *Eschrichtius robustus*. Right mandible in (*a*) lateral and (*b*) lingual views, and the left mandible in (*c*) lingual and (*d*) lateral views. Anatomical abbreviations: ag, alveolar groove; anp, angular process of the mandible; arp, articular process of the mandible; cp, coronoid process of either the mandible or scapula; gf, glenoid fossa of the scapula; mf, mandibular foramen; mef, mental foramina; sg, symphyseal groove of the mandible. See Pyenson *et al*. [28] and Peredo and Pyenson [29] for terminology. Original image credits: Courtney Johnson.

The skull of USNM PAL 706596 is nearly complete, represented by a disarticulated rostrum and an incomplete cranium with both mandibles (figures 2–4). The rostrum consists of the nearly complete right and left maxillae and premaxillae. Both premaxillae have perimortem breaks approximately midway along their lengths. The left maxilla is substantially more abraded particularly at the proximal end where skeletal elements are missing at the base of the rostrum (figure 2, table 1).

**Table 1.** Measurements of the cranium and mandibles of USNM PAL 706596 in cm (modified after [30] and [31]). Single asterisk indicates that measurement is estimated from articulating separate elements from the right side of the skull; double asterisk indicates that measurement is estimated from right side of the skull and multiplied by 2.

cranium	measurement (cm)	
condylobasal length	225.5*	
rostrum length	143.6*	
maximum width of mesorostral groove on the rostrum	21.5*	
width of rostrum at base	100.8**	
width of premaxillae at rostrum base	29.8*	
width of mesorostral groove at rostrum base	15.1*	
preorbital width	52.4*	
postorbital width	58.7*	
minimum distance between premaxillae anterior to bony nares	13.7*	
maximum width of premaxillae on cranium	27.5	
width of bony nares	13.4	
maximum width of nasals	33.4	
length of medial suture of nasals	17.8	
distance between lateral margins of premaxillae on vertex	22.3	
maximum length of frontals on vertex	29.4	
distance between anteromedial point of nasals and supraoccipital	41.6	
bizygomatic width	98**	
length of right orbit	12.1	
length of left orbit	N.A.	
length of right temporal fossa	15.2	
height of right occipital condyle	17.4	
width of right occipital condyle	8.9	
maximum distance between basioccipital crests	14.5	
width of occipital condyles	25.7	
distance between occipital condyles	10.4	
mandible	right (cm)	left (cm)
straight length	188	—
curvilinear length	194.4	—
height at distal end	13.5	13.1
height at 50%	19	—
height of coronoid process	20.5	20.6
length of coronoid process	5.6	5.6
length of mandibular fossa	33.0	—
height of mandibular canal	15	—
width of mandibular condyle	15.1	—
height of mandibular condyle	31.5	—

The cranium is missing the left squamosal and lateral half of the supraorbital process and antorbital process of the left frontal. Both nasals are intact (figure 2). Ventrally, the cranium is missing the vomer, both palatines, the left pterygoid and the left tympanoperiotic complex, and the right tympanic bulla is not preserved. The right petrosal is intact and articulated (figure 3).

Overall, the osteological surface condition of the cranial and rostral elements is lightly abraded and worn, but largely intact, probably attributable to its burial in beach sands and intertidal environments over a millennium (with possible exhumation and reburial events; see Discussion). There are also multiple cut marks on the lateral surface of the right squamosal (see more details in §4.5). In posterior view, the supraoccipital is intact, but with slight surface cracking in a line extending from the dorsal side of the foramen magnum to the right side of the apex of the supraoccipital shield (figure 2*b–d*). The left exoccipital is missing.

USNM PAL 706596 preserves both right and left mandibles, although the left mandible shows a perimortem break about midway along the body of the mandible (figure 4). The mandibles notably lack the prominent lateral curvature in balaenopterids and balaenids, and the coronoid process is low, not tabular, spatulate nor projecting; both traits are diagnostic for extant *Eschrichtius* (table 1).

#### 3.2.3. Appendicular skeleton

Both right and left scapulae are preserved for USNM PAL 706596. The dorsal margin of the scapula is even, forming a broad fan-shaped outline that is slightly more narrow than balaenopterids and closer in arc to balaenids (figure 4*e,f*). There is a robust coracoid process (relative to the overall size of the scapula) and a slightly convex glenoid fossa (table 2). The humeri, radii and ulnae from both sides are preserved, although the left humeral head is missing; the patent epiphyses on the latter elements are somewhat reflective of the early ontogenetic stage of this individual.

**Table 2.** Measurements of the appendicular skeleton of USNM PAL 706596 in cm (modified after [30] and [31]).

scapula	right (cm)	left (cm)
maximum height of scapula	74.3	73.2
width	52.7	52.2
anteroposterior length of glenoid fossa	15.7	14.7
transverse width of glenoid fossa	25.8	23.6
length of coracoid process	12.7	14.2
greatest width of coracoid process.	10.5	10.3
humerus	right (cm)	left (cm)
total length	31.5	31.0
transverse width of humeral head (without humeral head at proximal end)	18.0	(20.6)
anteroposterior width of humeral head	23.5	–
transverse width of greater tubercle	16.5	16.0
anteroposterior width of greater tubercle	18.2	17.4
anteroposterior width of shaft at midpoint	17.6	17.1
anteroposterior width at distal end	23.6	23.0
radius	right (cm)	left (cm)
total distal-proximal length	53.8	53.1
anteroposterior width at proximal end	14.1	14.0
anteroposterior width at midpoint	12.5	12.0
anteroposterior width at distal end	16.9	16.0
ulna	right (cm)	left (cm)
total length	52.4	52.2
length without olecranon	46.7	46.7
length of olecranon process	11	10
width at olecranon	15.1	14.7
width just below olecranon	9.2	8.7
width at midpoint	7.6	7.4
width at distal end	15	14.6

#### 3.2.4. Axial skeleton

**Figure 5.**
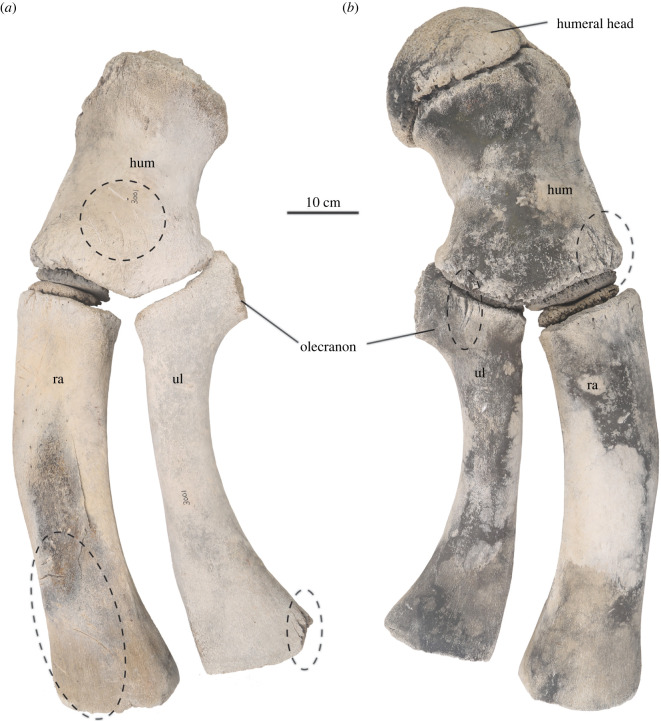
Humeri, ulnae and radii of USNM PAL 706596, *Eschrichtius robustus* for (*a*) left and (*b*) right side, in medial views. The dashed ellipses spotlight butchery marks. Original image credits: Courtney Johnson.

USNM PAL 706596 preserves nearly an intact series of vertebrae from the cervical to thoracic regions of the vertebral column (figure 5 and table 3). The vertebrae are well preserved, but post thoracic vertebrae are missing. USNM PAL 706596 does not preserve the atlas (C1), but the other cervical vertebrae (C2–C7) are preserved together. We have identified T1–T13 from the thoracic vertebral series; there is some uncertainty about the identity of T13.

**Table 3.** Measurements of vertebrae of USNM PAL 706596 in cm (after [32]). *L* is ventral centrum length. *W* is anterior centrum breadth. *H* is anterior centrum height. Asterisk indicates incomplete centrum.

vertebra	*L* (cm)	*W* (cm)	*H* (cm)
C2	4.3	28.4	10.7
C3	3.2	19.9	12
C4	3.4	18.2	12.5
C5	2.6	17.1	13.1
C6	2.7	16.6	13.4
C7	3.2	16.7	14.0
T1	4.5	16.6	13.5
T2	5.3	18.4	13.1
T3	6.0	18.2	12.8
T4	6.4	17.5	13.2
T5	8.0	17.4	14.3
T6	6.9	17.3	13.6
T7	7.9	17.0	13.7
T8	8.4	17.4	13.5
T9*	9.5	17.0	13.7
T10	9.4	18.2	13.8
T11	9.7	18.7	14.1
T12	9.3	19.2	13.9
T13	9.6	19.7	14.2

Along with the putative full series of thoracic vertebrae, USNM PAL 706596 preserves 11 ribs from the right side, of which seven ribs are complete and intact. We identified three left ribs, all of which are incomplete. We did not identify or locate any sternal elements (i.e. sternum).

### 3.3. Ontogeny and body size

**Figure 6.**
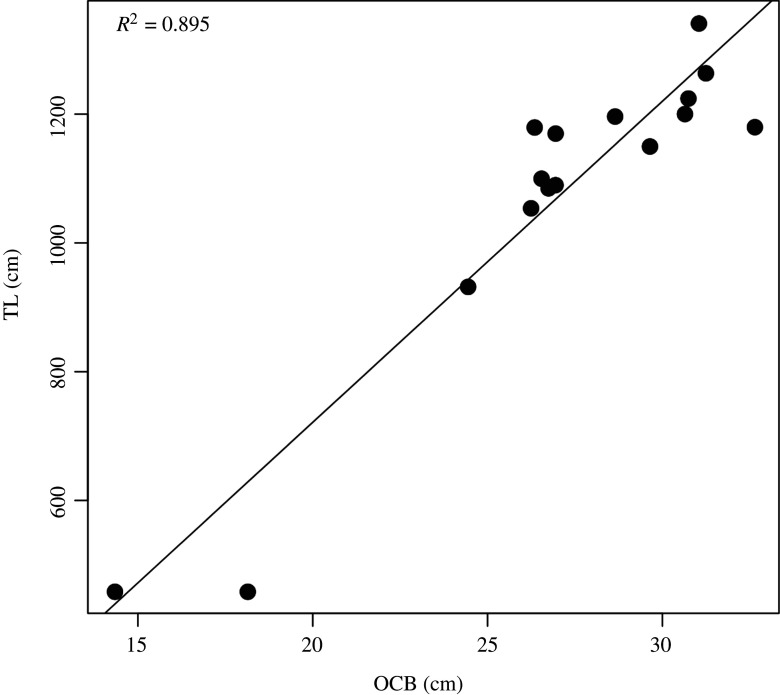
Estimating TL in grey whales using OCB as a skull proxy measurement. See electronic supplementary material, Text and table S3 for source specimen data from North Pacific grey whales.

**Figure 7.**
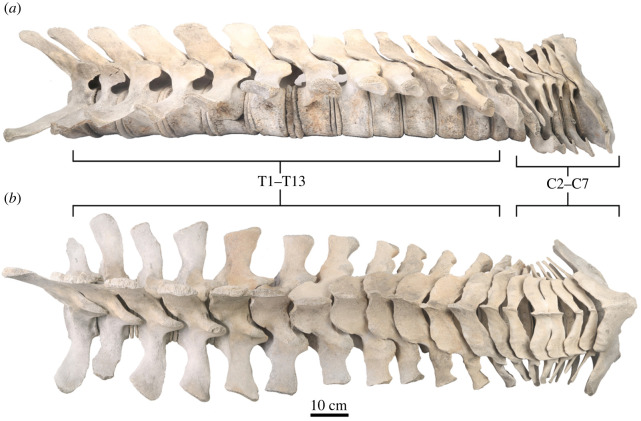
Vertebral series of USNM PAL 706596, *Eschrichtius robustus* in (*a*) right lateral and (*b*) dorsal views, with the entire vertebral column articulated in sequence, including epiphyseal discs on the vertebral centra. Original image credits: Courtney Johnson.

We assessed the skeletal maturity of USNM PAL 706596 using reliable osteological indicators for other baleen whales, such as the fusion of cranial sutures [33] and the textural surface of the occipital condyles [34]. For example, the open basioccipital-basisphenoid suture in USNM PAL 706596 (figure 2*a*) suggests the individual was physically immature at the time of death, although its occipital condyle breadth (OCB = 25.7 cm), a known body size proxy for cetaceans [34], falls within close approximation of other grey whale specimens that belonged to individuals with total lengths (TLs) between 9 and 10 m in life (figure 6). Based on a linear regression of *n* = 16 North Pacific grey whale skeletons, we calculate that USNM PAL 706596 had a TL of 10 m in life (*m* = 49.87, *b* = −276.06, *R*² = 0.895). Newborn grey whale calves are 4.9 m and sexually mature males and females are generally greater than 10–11 m in TL (see [35, pp. 46–51]; [12]). Together, these data suggest that USNM PAL 706596 was older than a yearling and close to sexual maturity, even though parts of its cranium and appendicular skeleton show a lack of suture fusion (figure 7).

Interestingly, the estimated TL (10 m) for USNM PAL 706596 is close to the body sizes of fossil grey whale specimens found at similar latitudes. Noakes *et al*. [12] and Garrison *et al*. [36] described late Pleistocene *Eschrichtius* mandibular and cranial material from Georgia and Florida, respectively, that belonged to individuals with TL between 9 and 10 m. Barnes & McLeod [37] reported a Pleistocene age *Eschrichtius* from the Palos Verdes peninsula of southern California uniquely represented by a complete skull, with an OCB equal to 27.5 cm, which similarly fits in the 9–10 m TL size class (figure 7). Together, this adds to the body of specimens found in the region [12,36] and suggests that USNM PAL 706596 may have been a young adult returning to lower latitude regions for breeding, as the extant Pacific grey whales do today.

### 3.4. Taphonomic description and butchery marks

Comparing the right and left sides of the specimen, the right side is better preserved than the left side. The bones from the right side of the body are more complete, less weathered, have better surface preservation and show evidence of root marking, probably from *Spartina* grasses abundantly represented in the tidal marshes (figure 4*b,e,f*). By contrast, the bones from the left side of the body are generally less complete, more weathered, have poorer surface preservation and rarely show evidence of root marking (figure 4*c,d*). This differential preservation pattern of the bones indicates that the individual grey whale skeleton was probably partially submerged in sediment on its right side, with the left side exposed to the environment. The level of overall preservation and the completeness of the skeleton probably resulted from the specimen's burial location. Barrier islands along the southeast coast of the USA change and migrate, particularly during storm events, which can rapidly bury stratigraphic layers. The depositional setting of USNM PAL 706596 may be somewhat analogous to that observed for relic saltmarshes on Georgia coast islands [38]. Additionally, the silt- and clay-dominated soil composition of the extensive marshes in this region would offer anaerobic, waterlogged conditions that can extend preservation.

**Figure 8.**
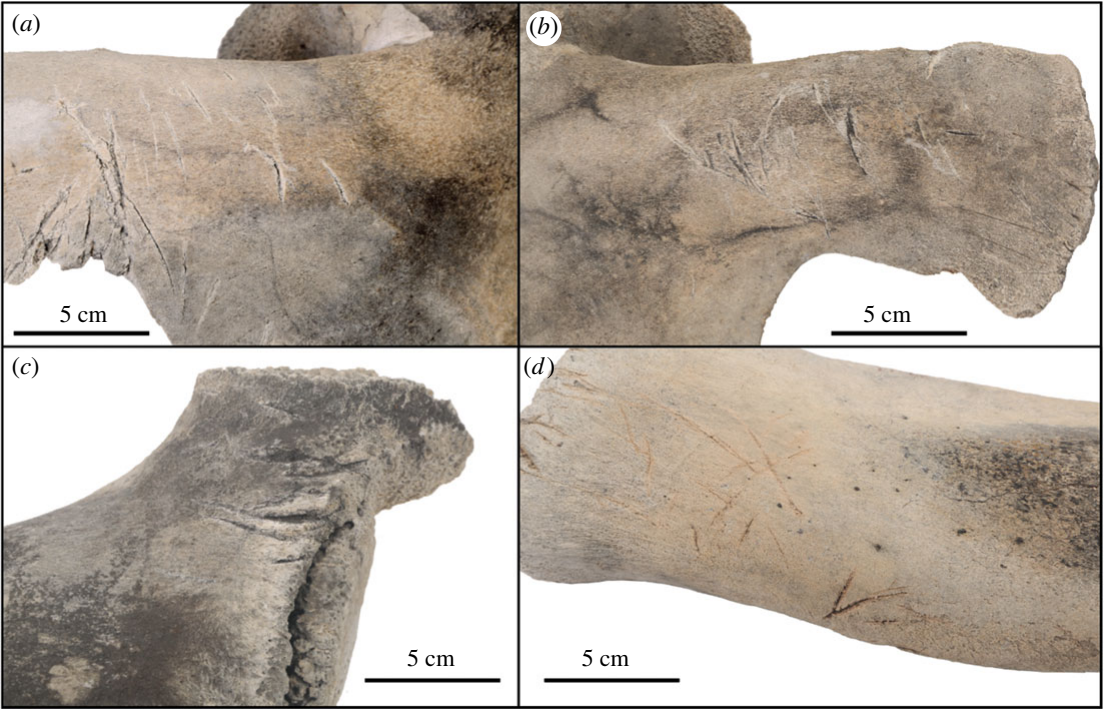
Skeletal elements of USNM PAL 706596, *Eschrichtius robustus* with butchery marks on the coronoid processes of the (*a*) right and (*b*) left scapulae, (*c*) the right ulna and (*d*) the left radius. Original image credits: Courtney Johnson.

Numerous skeletal elements have markings that appear to be butchery marks (figures 5, 8; electronic supplementary material, table S2). The size, shape, direction and consistency of the marks indicate they are much more likely to be butchery marks than marks inflicted by other large marine scavengers, such as sharks. Shark tooth marks on marine vertebrates, whether inflicted during a failed or successful predation event or during scavenging, leave distinctive and often serrated tooth marks which do not match these marks [39–41]. Additionally, terrestrial scavengers such as bears, wolves and cougars are not often observed scavenging on whales; their tooth marks include more rounded tooth pits (not observed here), elongated tooth scores (generally smaller in length and width dimensions than the marks observed here) and associated gnawing damage especially on long bone ends (also not observed here) [40]. Characteristic perforation and beak puncture marks by vultures, usually created on much smaller and thinner bones, were also not observed on this skeleton [42].

Some of the butchery marks exhibit typical features: they are straight and narrow, appear to have V-shaped cross-sections, are clustered, and the marks are often oriented in the same direction (e.g. most marks on both scapulae, right humerus, left radius and right premaxilla; see also figure 8*a,b*) [42]. Other marks are isolated or in smaller clusters but still exhibit typical butchery mark features (e.g. on right rib 7, posterior and right ulna, posterior; figure 8*c,d*). Still other marks are not typical butchery marks but are wider, more superficial and/or have a meandering or curved plan form (e.g. on right rib 5, anterior). Finally, some marks, such as those on the right squamosal (figure 2*f*), appear deeper, suggesting more bashing than cutting and raise the possibility that multiple types of tools may have been used in the butchering.

Many of the markings on the left side are less prominent due to the greater abrasion and decomposition of the skeletal elements on the left side. However, there is noticeable symmetry in the location and execution of the butchery marks across multiple skeletal elements (figures 5 and 8). The presence of so many markings on the right side suggests that the body was resting in a different orientation (perhaps more evenly on its ventral surface) during butchering that allowed easy access to both sides, before a shift to the right that resulted in greater submersion and preservation of the right skeletal elements.

## 4. Discussion

Historical accounts, fragmentary fossils, genetic sampling and habitat modelling have now contributed significantly to the portrait of grey whales in this basin. USNM PAL 706596 is the most complete record of a North Atlantic grey whale yet discovered and provides more information from a narrow window of time about the status of this species along the eastern seaboard of the USA.

### 4.1. Evolutionary history in the North Atlantic Basin

**Figure 9.**
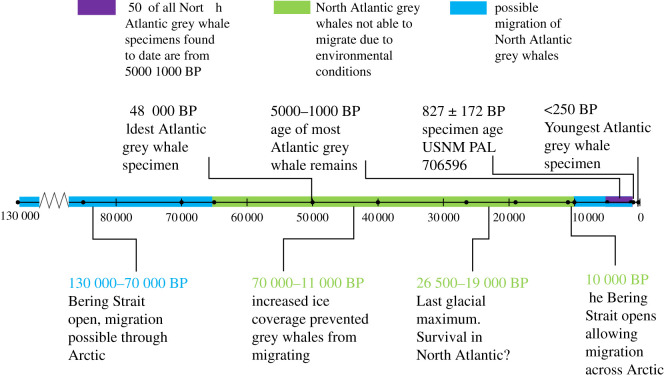
Overview of the Pleistocene to Holocene history of North Atlantic grey whales, along with major geological and environmental events.

While other baleen whale species have long periods of separation between Pacific and Atlantic populations since the Plio-Pleistocene (e.g. see divergence dates of *Balaenoptera* spp. in [43,44]), grey whales appear to have made multiple dispersals into the North Atlantic from the North Pacific. These intermittent exchanges, as evidenced by genetic similarity, maintained the Atlantic and Pacific populations as a single species, as originally suggested in the early twentieth century. Radiocarbon dating of the fossil and sub-fossil remains from around the North Atlantic largely fall into two temporal clusters of less than 10 kyr or more than 35 kyr (figure 9). Thus, evidence suggests that dispersal events into the North Atlantic occurred both in the Pleistocene and again after the opening of the Bering Strait between 10 and 6 kyr BP [13]. Although no fossil evidence has yet been found for persistence of the species in the North Atlantic during the Last Glacial Maximum (LGM), genetic diversity suggests that some portion of the population may have remained. (Any fossil sites of LGM age are probably far offshore on the continental shelf of the North Atlantic basin). Interestingly, one of the genetic lineages found in Holocene North Atlantic specimens shares a most recent common ancestor with Pleistocene specimens from the North Atlantic. Despite this evidence for persistence through the LGM, haplotype diversity of Holocene samples was probably significantly lower than Pleistocene diversity, with evidence for further declines in diversity (and thus probably also population size) in the mid-Holocene [13]. Alter *et al*. [13] offered that the timing of this decline could be related to the increasing ice extent diminishing ideal habitat in the North Atlantic and/or contributions from pre-modern whaling.

### 4.2. Historical-era whaling

Accounts in the literature indicate that some level of whaling on grey whales occurred in Europe and along the eastern seaboard of the USA [10]. Records from a 1611 English expedition to Spitsbergen state a lower quantity of oil in whales matching a description of grey whales yet a higher quality oil, demonstrating that it was a sought-after resource [10]. In his explanation of the ‘sandloegja’ whale in 1640, Guðmundsson described them as ‘good eating’ [45]. There are numerous accounts of a whale species resembling grey whales that was commonly found near shoals and sandbars and being beached or swimming up rivers and into harbours where they were pursued and hunted. In a description of early colonial settlements on Nantucket (pre-1672), the arrival of a grey whale into the local harbour and its successful catch was attributed to the start of the Nantucket whaling industry [10,46]. These and other anecdotes reveal that grey whales were certainly targets of Basque and early American commercial whaling, not surprisingly given their near shore range, but it is difficult to say the degree to which whaling contributed to their extirpation.

### 4.3. Archaeological evidence and context

From archaeological records, it is difficult to distinguish between systematic whale hunting versus opportunistic scavenging of stranded whales [14,22,47]. This is certainly the case with USNM PAL 706596 but additional context can be gained from evidence of human–whale interactions from other sites that pre-date or parallel this specimen. Systematic whale hunting has been an important part of coastal subsistence culture in the Pacific Northwest [21,47] and ‘outside of the Arctic, only Native North Americans in southern Vancouver Island and the Olympic Peninsula (of Washingon State) are definitively known to have whaled prehistorically’ [47, p. 256]. Interestingly, Wellman *et al*. [47] note whale remains from the Par-Tee site on the Oregon coast dated from 350 BC to AD 1150. They observed fine cut marks and gouge marks on a mandible, scapulae, a humerus, ulnae, one vertebrae, ribs and phalanges assigned to grey and humpback whales. Despite a previous analysis which identified an embedded point in a humpback phalange from Par-Tee [48], Wellman *et al*. [47] conclude that the lack of definitive strike marks on the bones of whales found at Par-Tee reflects that whales were largely opportunistically hunted and that beached and/or drift carcasses were scavenged. Evoking an explanation that matches the range of butchery marks for USNM PAL 706596, Wellman *et al*. [47] propose that the butchery at Par-Tee indicates **‘**processing for meat removal and oil extraction rather than specialized modification and utilization of certain elements' [47, p. 272].

Monks [21] presented a zooarchaeological analysis of pelagic whale remains butchered by Nuu'chah'nulth (Nootka) people on the northwest coast of North America from Ozette in Washington State (where deposits date from the past 2000 years) and five sites in the Toquaht area on Vancouver Island (where deposits date from the past 4200 years) in British Columbia, Canada. Also relying on ethnographic data and a previous study by Fiskin [49], Monks [21] noted that the Nootka people primarily used whales as sources of blubber and oil, as well as meat, bone, viscera, internal fat and sinew. Butchery marks including cut marks, chop marks and gouge marks on skulls, rostra, mandibles, humeri, radii, ulnae, scapulae, vertebrae, phalanges, ribs and a hyoid; several bones were modified into cutting boards and other artefacts (at Par-Tee, whale bones were also made into numerous artefacts; [47]). Many of the Nootka butchery marks were interpreted as resulting from oil extraction (also consistent with evidence of charring), or meat and sinew removal; few bones exhibited butchery marks associated with disarticulation. Monks [21] attributed the general cuts found on the humerii, skull, thoracic, lumbar and caudal vertebrae to oil extraction, although it is not clear how prevalent this method may have been across whale species or peoples. Nevertheless, the elements for oil extraction and corresponding butchering marks (on the distal anatomical surfaces) on USNM PAL 706596 are consistent with Monks [21]'s oil extraction explanation. Monks [21] described a tentative scheme for interpreting primary and secondary butchering, presumably after disarticulation. The implements inferred to have been used include fine slicing and incising tools, coarse cutting tools, chopping and gouging tools, and smashing tools.

The presence of eastern Atlantic grey whales at other zooarchaeological sites has been recently demonstrated with advancements in ancient DNA barcoding and collagen fingerprinting methods from fragmentary remains with limited diagnostic traits (see [14] for a review). Using this pairing of methods, Rodrigues *et al*. [17] identified grey whales from pre-Roman and Roman sites (ranging from 400 BCE to 450 CE) in northern Spain and the region surrounding the Strait of Gibraltar in the Mediterranean Sea, arguing that grey whales entered the Mediterranean to calve at this time. Rodrigues *et al*. [17] also noted how these occurrence data parallel historical accounts that suggest sufficient population density, along with a Roman salting infrastructure that could have supported a whaling industry that targeted grey whales. These methods have also identified northern European records for grey whales from 1000–2000 CE, including vertebrae and ribs from Scotland [50,51] and Norway [18]. While the Norwegian specimen gives no indication of human interaction, the Scotland specimens show significant modification for human use.

At late Roman and early Medieval sites near London, van den Hurk *et al*. [22] found no association with grey whales among cetacean butchery material; instead they observed a chop mark on the scapula of a likely North Atlantic right whale (*Eubalaena glacialis*) dated to 1050–1150 CE; seven cut marks on a skull fragment of a common minke whale (*Balaenoptera acutorostrata*) dated to 1150–1350 CE; a saw mark on the rostrum of a different common minke whale dated to 1200–1500 CE; several chop marks and cut marks on the proximal end of a mandible of a large baleen whale dated to 0–1600 CE. On the Medieval (400–1600 CE) Dutch and Belgian coast, van den Hurk *et al*. [52] did, however, identify five grey whale specimens, arguing that grey whale exploitation may have been rather frequent. Archaeological evidence from middens suggests rural use of grey whales beginning in 400 CE, probably originating from opportunistic strandings, then extending to elite and ecclesiastical consumption in the eighth century with the development of active whaling in the twelfth century [52]. Overall, grey whales appear to have persisted in Europe well into the seventeenth century, although it is unclear if the low abundances of their remains at zooarchaeological sites reflect secular patterns of ecological abundance, a legacy of whaling, their **‘**invisibility’ in the archaeological record or some combination of these factors.

Subsistence information from archaeological sites indicates that people lived along the North Carolina coast in the Middle (approx. 300 BC–AD 800) and Late (approx. AD 800–1650) Woodland periods. The latter encompasses the time of contact with Europeans and a shift to agriculture such as maize, including a variable diet with terrestrial resources and wild wetland and estuarine resources including fish, birds, turtles and shellfish such as oysters, clams, whelks and crabs [53,54], either seasonally or year-round [55]. The Woodland period includes the first use of the bow and arrow, with continued use of spears and atlatls with stone projectile points, as well as pottery for cooking and storage, while copper was mainly used for ritual objects [56,57]. There is also some evidence of potential ritual use of whale bone from the southeastern USA well before this time; for example, the cremated human remains of multiple individuals were found buried in the direct centre of a circular shell midden including a copper artefact and the vertebra of a pygmy sperm whale (*Kogia breviceps* [58]) at a Late Archaic (approx. 4000 cal BP) site in coastal Georgia [59]. Based on the geographic location and age of USNM PAL 706596, its Gregorian calendar date of 1194 ± 172 years CE indicates that the marks on this specimen were made by stone rather than metal tools.

Given the radiocarbon age, appearance of the butchery marks, and location where the specimen was found, it seems likely that USNM PAL 706596 represents the result of an opportunistic butchery event, not the remains of a systematic hunt. In contrast with aforementioned accounts from other regions, it is interesting to note that the skeleton of USNM PAL 706596 was left so intact and not much bone was collected for fuel or other uses. Additionally, the skull was not butchered; grey whale specimens from 4 to 8 kyr BP from The Netherlands had holes in the occiput above the condyles in order to remove brain tissue [8,60]. Thus, this specimen seems to have been processed rather opportunistically, removing mainly flesh and muscle. Given the symmetry of the butchery marks and placement of the cuts at joints, however, the marks were probably inflicted by at least somewhat experienced butchers that perhaps had processed large whales previously. In many ways, the age and taphonomic traits of USNM PAL 706596 parallel those of a Southern elephant seal (*Mirounga leonina* [61]) specimen recovered from a Holocene river site in Indiana [62]. While an extralimital record, its butchering marks raised similar questions and probably also reflected an opportunistic event.

### 4.4. Ecological context

Based on its size and osteological traits, USNM PAL 706596 was close to sexual maturity when it died. Previous studies (e.g. [12,36]) have found evidence to support that the southeastern coastline of the USA, from North Carolina to Georgia, was probably a breeding ground for this population, as it is today for North Atlantic right whales (*Eubalaena glacialis* [63]). Grey whales along the North Atlantic coastline probably paralleled latitudinally and environmentally the shallow bays and lagoons that North Pacific grey whales select off Baja California, Mexico for breeding and calving in the winter months. The habitat where USNM PAL 706596 was collected is a shallow, tidally influenced area at the mouth of the New River, in North Carolina. The individual may have gotten disoriented or died offshore and washed into the marsh area. Regardless, the presence of an immature individual further supports the idea of these coastal habitats as breeding and calving grounds for North Atlantic grey whales.

### 4.5. Future work

Only three western North Atlantic grey whale specimens have previously been analysed for genetics (Alter *et al.* [13]). Ancient DNA sampling and collagen fingerprinting of USNM PAL 706596 could shine additional light on potential population structure. Given what we know of baleen whale and even grey whale migration and population structure today, it seems very feasible that separation between eastern and western populations may have, in fact, existed during multiple pre-LGM episodes at or below current sea-level. While small sample sizes for the North Atlantic basin inevitably result in a partial picture, additional sampling of western North Atlantic specimens, such as USNM PAL 706596**,** would be helpful for further characterizing the overall diversity of Holocene grey whales in the North Atlantic. Additionally, if this specimen shares a common haplotype with Pleistocene specimens, it may provide additional insight on persistence of the species in the basin through the LGM. Finally, the extent of skeletal elements in USNM PAL 706596 can provide many opportunities for further comparative osteological and archaeological work both within the North Atlantic basin specimen pool and against modern and fossil North Pacific grey whale specimens.

## Data Availability

The specimen, USNM PAL 706596, is housed in the Smithsonian Institution's National Museum of Natural History and is available for public research access. The data are provided in the electronic supplementary material [64].
